# Antimicrobial resistance induced by genetic changes

**Published:** 2009-04-25

**Authors:** BI Coculescu

**Affiliations:** Microbiology, Parasitology and Virology Laboratory–Centre of Prophylactic Medicine, BucharestRomania

## Abstract

Decoding the mechanisms of resistance to antibiotics is essential in fighting a phenomenon, which is amplifying everyday due to the uncontrolled excessive and many times unjustified use of anti–microbial substances. At present it has become a matter of public health, together with the resistance of Mycobacterium tuberculosis to tuberculostatic or the spreading of the AIDS virus which not only affects the European countries but the entire globe.

This paper presents the genic mutations taking place at the level of bacterial chromosome and inducing the resistance to antibiotics.

## Introduction

Microbial resistance to antibiotics, a process that has known a rapid uncontrolled growth during the last two decades in the entire world, is widely accepted today as one of the major problems of public health at the world's level [,^2^,^3^]. It manifests itself by the seriousness of infections or the prolonged clinical symptomatology duration, increase in the number of hospitalization days, and, last but not least, by the resulting costs [4].

The period between the beginning of the antibiotics treatment (the 1940s) until the emergence of bacteria expressing efficient mechanisms of resistance is too short (50–60 years) to explain the coming into being and spreading of the resistance genes only through the phenomenon of spontaneous mutation [5,6].

The solutions to the problems of anti–microbial resistance are a direct consequence of understanding the mechanisms at the basis of its emergence. That is why the genetic molecular mechanisms resistant to antibiotics must be known in order to successfully fight the resistant or multi–resistant bacteria (MDR–‘multidrug resistance’).

We frequently refer to bacteria as being resistant to antibiotics but we seldom know what that means [5]. In the expert literature, the definitions are more of the notion of resistance to antibiotics. There is resistance for a microbial population when the medium concentration of *in vitro* inhibition is bigger than the concentration, which can be achieved at the place of the *in vivo* infection [7].

Even the most resistant bacteria can be inhibited or killed by a high enough concentration of antibiotics. However, not all the patients can tolerate very high bacteriostatic concentrations with a bactericide effect, which would be needed in such cases. Bacterial species widely vary in their susceptibility to an antibiotic. For example, in the United Kingdom, most strains of the *Streptococcus pneumoniae* are inhibited by a minimum inhibitory concentration (CMI) of 0.01mg/L benzilpenicilline, while the inhibition of the growth and multiplication of the *Escherichia coli* strains a CMI of 32–64mg/L of the same antibiotic, but this level cannot be reached in the human body [5].

From the bacteriological point of view, Decoster and collab. consider that a strain is resistant when it can develop in the presence of an antibiotic concentration higher than the concentration that inhibits most of the strains belonging to the same species [8].

More mechanisms have developed into bacteria gaining resistance to antibiotics. These mechanisms can either chemically transform the antibiotic or inactivate it by removing it completely from the cell, or by modifying the aimed locus so that the latter can no longer be recognized by the antibiotic [9,10].

The DNA of the bacteria, no matter what their chromosomal, plasmidic (episomal [11]) or phagic nature is, may include genes encoding the mechanisms of resistance to drugs, such as the inactivating enzymes of the antibiotics, pumps of efflux, modification of the antibiotic action target etc. [5,9,10].

Bacterial resistance towards antibiotics can be natural (intrinsic, innate) or acquired by mutating the endogenous genes or by incorporating of the exogenous genes of resistance [5,6,8,10].

## Natural resistance

Bacteria can have a natural resistance to an antibiotic, meaning they can grow and multiply in the presence of maximum concentrations of antibiotics tolerated by the body, their development not being influenced by that drug in any way. For example, certain microorganisms, such as the anaerobes, lack a transport system for an antibiotic, thus being intrinsically resistant to aminoglycoside. Another kind of organisms, i.e. mycoplasmas–without a cellular wall – lacks the aim for the antibiotic and thus they are naturally resistant to the action of the beta–lactamic antibiotics for the blocking of the cellular wall synthesis, as the latter do not possess the enzymes which link the penicillin (PBP). In the case of the Gram-negative bacteria, the peptidoglycans stratum is covered by an exterior membrane which plays the part of a hardly permeable barrier especially towards the penetration of antibiotics. For example, due to the fact that the glycopeptidic molecules are big (vancomycin–1448Da and teicoplanin–1900Da), they cannot penetrate the external membrane of the Gram negative bacteria's wall, therefore the peptidoglycan is not accessible to the action of these antibiotics. Similarly, penicillin G, rifampicin, novobiocin, fusidic acid or the macrolide antibiotics (erythromycin) do not penetrate the external lipopolysaccharidic membrane of the enteric Gram negative bacteria (*E. coli*)[6,10,12].

Certain pathogenic organisms, such as *Pseudomonas aeruginosa, E. coli, Enterobacter cloacae*, have low permeability of the external lipopolysaccharidic membrane, thus constituting an obstacle in the diffusion of antibiotics inside the bacteria, consequently such organisms show natural resistance to a lot of antibiotics [6,9]. For example, the *Ps. aeruginosa* has a natural resistance to ampicillin and tetracycline, *E. coli* to vancomicyn etc. [5]. *Ps. aeruginosa* is naturally resistant to certain antibiotics such as aminopenicillin, cephalosporin of the first and second generation or kanamycin, due to the synthesis of beta–lactamase associated with a low permeability of the cellular wall and a mechanism of active constitutive efflux [5,15,16]. It is worth noticing that the presence and/or the acquiring of a single mechanism of resistance do not always confer resistance to an entire class of antibiotics and, consequently, crossed resistance is not always a rule. For example,*P. aeruginosa*, resistant to kanamycin stays sensitive to resembling antibiotics (gentamycin) [6,17].

Natural resistance is genetically supported by the **bacterial chromosome** [6,8]. The bacteria that synthesize antibiotics have to protect themselves from the antibiotic that they produce [6,10]. For example,*Streptomyces* show an intrinsic resistance to the antibiotics they produce, as a mechanism of self-protection from other organisms tending to consume the nourishing resources in the environment [13]. In *Klebsiella spp., Enterobacter spp., Serratia spp., Morganella spp., Providencia spp., or Ps. aeruginosa*, resistance is determined by the production of a chromosomal beta–lactamase of the cephalosporinase type that inactivates the beta–lactam antibiotics [14,15]. It has been noticed that the genes that are resistant to antibiotics are frequently localized by restriction fragments adjoining those responsible of producing the antibiotic.

*Serratia marcescens* naturally produces an enzyme codified by a chromosomal gene capable of inactivating the kanamycin, tobramycin, netilmicin and amikacin, and Providencia stuartii produces an enzyme capable of inactivating neomycin, gentamicin, tobramycin and netilmicin [6].

Anaerobic species belonging to the *Bacteroides genus* and being part of the human digestive duct microbiota are naturally resistant to aminopenicillin and numerous cephalosporins with a wide spectrum producing codified beta–lactamase of chromosomal. The species *Staphylococcus saprophyticus* is naturally resistant to phosphomicine [6].

The codifying genes of the resistance factors existed before the moment of introducing the antibiotics in clinics as they were recognized in the bacteria collections created before the use of antibiotics [5,6]. For example, it was discovered that a strain of *S. aureus*, isolated previously to the discovery of antibiotics, synthesizes the beta–lactamase. It is considered that the lysozyme (muramidase) in the nasal secretion exercised a selective pressure favoring the selection of the cells resistant to antibiotics [6,18]. The clavulanic acid (produced by the *Streptomyces clavuligerus*) is a natural inhibitor of the beta–lactamase, conferring resistance to beta–lactamic antibiotics. The co–production of cephamycin (a beta–lactamic antibiotic) and clavulanic acid is constant, meaning that there are no known producers of clavulanic acid that do not produce cephamycine [6,9,19].

In enterococci (except the *E. faecium* and *E. durans*) natural resistance manifests in lincosamides and streptogramin A (dalfopristin), consequently this type of resistance can be used as an orientation test for identification [6, 20,21].

Nevertheless, most of the bacteria become resistant to antibiotics through one or more genic mutations that are further presented in the article.

## Acquired resistance

The genic bacterial apparatus is represented by two types of structures: the nucleoid, which, from the structural functional point of view corresponds to the chromosome, and a second category of structures is represented by the extrachromosomal genic elements (plasmids, transposons, integrons, sequences of insertion etc.)[6,9,11, 22].

According to the two types of structures, the genic determinants are divided in two categories [6]

- essential genes (euchromosomal), situated in the structure of the chromosome and bearing the genetic information that ensures the development of the essential functions for the existence of the cell, meaning the set of the minimally necessary determinants to encode the architecture of the cell and to ensure the energetic and biosynthesis metabolism, growth, division and regulation of different cell activities, and
- accessory genes, with plasmidic localization or in the structure of the transposable genetic elements or phages bearing the genetic information that allows the cell to acquire a better adaptation to new or modified environment conditions.

For example, after the complete cartography of the genome *E. coli K_12_*, it resulted that the latter is made of about 4,400 genes, but for the growth of the bacteria in the laboratory only a few hundred would be necessary [23, 24].

Most of the naturally sensitive bacteria to antibiotics become resistant due to certain genetic chromosomal changes (in 10–20% of the cases) or extrachromosomal (less than 80% of the resistance cases), a process that is called genetic resistance [8,22].

The acquired antimicrobial resistance emerges by the selection, consequently to the exposure to antibacterial drugs (for example, in medicine, agriculture or veterinary practice), of the species naturally resistant or due to the emergence of variants resistant inside some previously sensitive species [5].

The exposure to antibiotics is not the cause in itself for the manifestation of the bacterial resistance to drugs. The bacterial changes allowing the bacteria to resist to antibiotics naturally appear as a consequence of the mutation or as a result of the genetic combination [5,8,9].

Bacterial resistance to antibiotics was recognized immediately that the first antibiotics had been introduced in clinics [6,10]. In 1943, in certain strains of Stafilococcus aureus, there was discovered the resistance to penicillin G, an antibiotic that had started to be used since 1941, and two decades later, in 1962, two strains of *S. aureus* were made evident for the first time, and they were resistant to methicillin that had been introduced in therapy since 1961 [22]. In fact, it has been ascertained that the emergence of resistance is inevitable after the introduction in the market of a new antibiotic [10], and that was confirmed over the years by the current medical practice. For example, in 1962, ampicillin appears, and in 1964 the resistance of the enterobacteria to the drug is emphasized, or in 1980 cephalosporins started to be commercialized, followed in 1981 by the development of the resistance of the enterobacteria to the previously mentioned anti–microbial substances [22]. Consequently to the studies entered upon it has been noticed that the proportion of the bacterial strains resistant to antibiotics varies depending on the country, the species of the organism and the used antibiotic–victim to its own therapeutic success [25,26,27,28,29].

Such genes that encode the resistance to antibiotics have existed in the natural environment of selection of the bacteria even before the use of antibiotics in therapeutic practice [5,6] (1941–penicillin G, discovered in 1929 by Sir Alexander Fleming [10]), determining the capacity of a species strains to grow and multiply in the presence of maximum concentration of antibiotics without any major risks for the human body.

The notion of gene was proposed by WI Johannsen (1909) in order to determine the basic hereditary unit, which is localized in chromosomes. The gene is the unity function of the genetic complex controlling a phenotypical character. TH Morgan, in 1911, defines gene as being the function unity, recombination, and mutagenesis, which does not have subdivisions [30].

The present conception states that gene is defined as being the poly–nucleotide segment of the DNA molecule holding the genetic information necessary to the synthesis of a polypeptidic chain with specific structure and function. In other words, the term of gene stays associated with the unity of genetic function and represents the sequence of nucleotides in the DNA, capable to be translated into the sequence of amino acids of a protein [30].

An essential part in the emergence and development of the bacterial resistance to antibiotics is played by the genetic variability. It is the result of one of the following events: genetic mutation, the transfer of genetic material between microorganisms by transformation, transduction, or conjugation and transposition respectively.

The genetic mechanisms implied in the acquired resistance can be the mutations affecting the genes present on the bacterial chromosome.

Mutations are sudden changes in the genetic material (dowry), transmissible and definitive (they stay ‘stable’) in the succession of generations, for a character or group of hereditary characters, as a reply to the action of certain modifying factors (endogen or exogenous) [30].

In prokaryotes, including the bacteria, which reproduce by plain mitosis, any mutation is transmitted to the descendants (vertical transmission).

As a definition, by genetic mutation we mean any disturbance in the sequence of the genetic code, susceptible to induce the synthesis of a protein with a flaw (abnormal biological structure and function).

Genetic mutation is an accidental change in the sequence of the polynucleotides of a gene; it affects more nucleotides of a sequence or it is limited to only one nucleotide (in this case we deal with a punctiform mutation) of one or both catenae of a DNA molecule. Mutation appears as an unpredictable error in the replication of the DNA. The different forms of a gene appeared by mutation are called allele and they occupy the same genetic locus as the original gene [30].

Reported to the size of the change, mutations can be: punctiform or extended.

By the modality of their appearance, mutations can emerge under conditions considered as normal(natural)–in this case, they are called spontaneous mutations, or they can emerge consequently to mutagenic agents – induced or artificial mutations. Subject to the action of certain physical agents (UV radiations, ionizing radiations, visible light, warmth etc.) or chemical ones (alkyl agents analogue to the nitrogen bases–polycyclic aromatic hydrocarbons like bensopiren, nitrogenous acid), named mutagenic agents, the frequency of the replication errors of the DNA grows, consequently leading to the growth of the induced mutation rate [30].

Mutation has spontaneous character and it can appear in any cell (prokaryote or eukaryote) at anytime in the cell's life.

Generally, the bacteria situated in the stationary stage of the growth line show a single circular chromosome of quite variable size [31]. In the dividing bacterial cells that contain more chromosomes, mutation is produced at the level of one of the chromosomes only, usually a single gene being involved, the other homologue genes not being affected by the mutation, and the modification of the nucleotide sequence initially shows just on one of the 2 complementary catenae of the DNA chain.

Regardless of the conditions under which they appear, spontaneous or induced, mutations have an unpredictable character meaning they are not specific for a certain genetic locus [30].

The ratio of mutation is the probability of the apparition of a mutation. It is measured by genetic events per cell and per cellular generation. It is very reduced, about 10^–9^, and for two characters, 10^–18^. In other words, the probability of the apparition of a mutation for one character is 1 to each 10^9^ cellular divisions. The mutation ratio grows as subject to the action of the mutagenic agents. In addition, it grows considerably after mutations of genes specifying the enzymes implied in the replication and repairing of the DNA [30].

Under those circumstances, certain genes of resistance have appeared by accidental mutations that offered a selective advantage to the carrying cells [10].

The frequency of mutation is defined as being the proportion of a certain mutant in a cellular population. With bacteria, mutations have a frequency of 10^–5^–10^–9^, without being determined by the environment, which only plays a part in the selection of the mutants. Thus, the frequency can grow as a consequence of the selection [30]:

- relative selection is produced when the mutant has a time of generation shorter than that of the parental population;
- absolute selection is achieved by an environment factor favorable to the development of a mutant, yet unfavorable to the parental population. For example, subject to the action of a certain antibiotic, the resistant adequate favored mutants multiply and generate a resistant clone while the parental sensible population is eliminated.

Accordingly, the frequency of the spontaneous mutation for the resistance to antibiotics is of approximately 10^–8^–10^ –9^ meaning that in an infection treated with antibiotics, one at each 10^8^–10^9^ bacteria will develop resistance by the process of mutation. In the strains of *E. coli* it has been estimated that the resistance to streptomycin is acquired at a rate of approximately 10^–9^, when those are exposed to high concentrations of streptomycin [10].

Punctiform mutations (micro evolving genetic modifications) in the structure of the chromosomal genes can modify the sensibility of organisms to antibiotics by structural modification of the target. For example, some microorganisms modify their beta–lactamase as a consequence of a punctiform mutation and thus the scope of the enzyme extends. Similarly, the mutational change of just one amino acid in a protein of the ribosomal subunit 30S determines the resistance of the cell to streptomycin [6]. It is about a mutation in the structure of a codifying gene for the small ribosomal unity 30S. Streptomycin does not have any relation with this subunit. If the latter stays functional, a form of resistance has been obtained [32].

There are five mechanisms of the genic mutation: substitution, deletion, addition, inversion and duplication.

- (1) Substitution–replacement of a nitrogenic base (so called nucleotide base) with another–in its turn it can be [30]:
  - a)Substitution by transversing emerges when a purine base (adenine–A–, or guanine–G) of a catena is replaced by a pyrimidine (thymine–T–or cytosine–C), or a pyrimidine one with a purine one [33]. This mutation not only affects the codon (the base triplet in the polynucleotide) where the transversing has been produced.
  - b)Substitution of a nitrogenic base (purine base: A, G, or pyrimidine: T, C) by analogue compounds of it (5 bromouracil or fluorouracil) which leads to the alteration of the complementary nucleotide couple. The result is the emergence of a protein with a punctiform structure flaw, different from the old one by the respective new amino acid introduced in its structure.
  - c)Substitution by transition, when a purine base of a catena is replaced by another purinic base (i.e. A → G) or a pyrimidinic base is replaced by another pyrimidinic base (T → C) [33], changing the complementarity of the nucleotides.
- (2)Deletion (suppression) of a base in the structure of a gene affects all the codons (triplets) following the point of nucleotide suppression.
  As a direct result of the elimination of a nucleotide, a protein with a longer or shorter sequence of amino acids. Thus, if this mechanism (deletion) transforms a triplet with ‘meaning’ into a nonsense triplet (terminator codon) then the sequence of amino acids will be much shorter than normal. Or vice versa, if the terminator triplet (nonsense) is transformed into a triplet that codifies an amino acid, then the sequence will be much longer than normal [30].
- (3)Addition (insertion) of a supplementary nucleotide in the structure of a gene affects all the codons following the insertion point.Deletion followed by insertion affects those codons situated between the deletion point and the insertion point. The codons following the insertion point will have normal ‘structure’.
- (4)The inversion of a codon of a gene structure has a punctiform effect. For example, the CCA codon, which codifies proline, by inverting the order of the ACC bases will codify histidine. The inversion of a nucleotide with another nucleotide, thus modifying a codon, has similar consequences with those of substitution.
- (5)Mutation by duplication–deficiency leads to the formation of new proteins that can be different from the old ones: either the two genes associate in order to codify a single protein, the sequence of which will be a duplication of the original protein, or the two genes (created by duplication) will function at the same time so that the production of proteins can be modified.

The vulnerability of the DNA molecule towards chemical factors (alkyl agents, nitrosating agents, structural analogues) and physical (UV radiations) creates the premises for the emergence of DNA ‘injuries’, which generally consist of [34]:

- purine degradation;
- breaking of the phosphodiesteric links;
- cross–linking of the bases to the opposite catenae;
- change of the tautomeric conditions which leads to the possibility of an erroneous coupling of bases, either in the course of replication or by substituting a base belonging to DNA by a structural analogue; that is how, due to the fact that 5 bromouracil (an analogue of thymine) prefers the enolic form instead of the cetonic one, adopted by the thymine that couples with the guanine not with the adenine, which leads to the replacement of the pair A=T by G=C (transition);
- spontaneous deamination or subject to the action of the nitrogenic acid, followed by the formation of compounds that either do not couple with the nitrogen bases or they are predisposed to erroneous coupling. Thus, by deamination, the adenine leads to hypoxanthine, guanine to xanthine, and cytosine to uracil, products that are not bases belonging to the DNA and they will be recognized and removed by the reparation enzymes;
- dimerization of thymine, followed by cyclobutanic actions within the same catena;
- insertion of one or more supplementary bases. For example, when cellular cultures are treated with acridine, the latter interferes between two bases of a chain. During replication, on the complementary catena, to the acridine, a supplementary base inserts itself and links covalently. At a future replication there will be a supplementary pair of bases;
- deletion of one or more bases. The phenomenon means hydrolyzing a base of the chain, which is possible when the pH and temperature vary, or by the action of agents that modify a base in such a manner as to make it stop being complementary to any other base. By replication, the ‘void’ appears on both catenae.

These injuries of the DNA molecules can be corrected by two repairing mechanisms that detect the injury and repair the injured catena: one repairing constitutive system by excision–resynthesis, and a second one, the inducible system of repairing–the SOS system–represented by a series of factors of protein nature; their fidelity in replication is just approximate, hence its name of‘reparatory system subject to errors’. It is important to notice the fact that each time a catena is injured, the other will not only serve to keeping the genetic information but to repairing the injured catena as well [11,34].

The injuries escaping the repairing systems become mutations [34]. The errors of the replicative synthesis of the DNA and the inability of the systems to repair the DNA lead to a frequency of spontaneous mutation of a pair of bases/10^7^–10 ^10^ cells, meaning that for each 10^7^–10^10^ cells, only one base suffers modification. Anyway, the rate of spontaneous mutation generating resistance is lower, as for the emergence of primary resistance multiple mutations are needed. That is why it has been considered that the emergence of the strains resistant to antibiotics by mutational processes, during therapy, is not probable [6].

The expert literature describes numerous cases of bacterial resistance to antibiotics, usually as a consequence of the apparition of one or more spontaneous mutations.

Thus, some bacteria including *Mycobacterium tuberculosis* or *Staphylococcus aureus* resistant to meticilline, can acquire resistance to rifampicin by a punctiform mutation of the beta–subunit of the RNA polymerase depending on the DNA, a mutation codified by the rpoB, which leads to the loss of specificity for the molecule of rifampicin. [35,36,37,38]. Consequently, the RNA polymerase will no longer show affinity to rifampicin and it will no longer be affected by the inhibiting effect of the antibiotic. The mutations induced by the rifampicin are produced in the gene codifying the synthesis of the bacterial RNA polymerase; these events usually appear in the 3 short regions highly preserved of the subunity beta forming the area known as ‘the determined region of resistance to rifampicin’, comprised between the remains 505–534 (in *E. coli*), at a distance from the active locus of the enzyme. In mycobacterium, over 90% of the mutations are due to the modification of one nucleotide of the codifying gene of the beta–subunity. For*Mycobacterium tuberculosis*, the mutations conferring resistance to rifampicin are most frequently found in the codons 531, 526, and 516–in descending order of the frequency [39] – while in *Staphylococcus aureus* the mostly identified mutation is in codon 481 [40].

The genetic analysis established that the spontaneous resistance to fluoroquinolone (such as ciprofloxacin or norfloxacin) can be the result of a punctiform mutation in each of the two genes *gyrA* and *gyrB* that codify the two protein subunities of an enzyme, DNA gyrase, fact that leads to enough conformational modifications of the gyrase, so that the affinity for fluoroquinolone is diminished or absent [41,42]. In a study performed in China in 2007, Li–Fen Hu et al. underlined the fact that that the most common mutation met in 20 isolated bacteria of Shigella  resistant to fluoroquinolone (47.62% of the cases) were in codon 83 of the *gyrA *(transitions TCG → ATC and TCG → TTG), resulted from the replacement of the serine by isoleucines and leucine [43]. In fact, the same conclusion was previously reported by Dutta et al. in 2005 [44].

Resistance to streptomycin may appear due to the mutations at the level of the genes that codify 16S rRNA, thus reducing the affinity of the antibiotic for the molecule 16S [45].

The loss of activity of the enzyme NADPH nitroreductase–that activates, at intracellular level, the metronidazole in order to have antimicrobian effect–can be the result of a nonsense deletion or a mutation in the rdxA gene [46,47]. Moreover, the activity of the NADPH nitroreductase could be dramatically reduced  by a single spontaneous mutation (the change of one amino acid) occurred in the strains of the *Helicobacter pylori* sensible to metronidazole, a fact that reduces its capacity to activate the substance belonging to the category of nitroimidazoles. All of these mutations have as a result the loss of the enzymatic activity necessary for the drug to be able to act inside the cell, consequently the bacteria become resistant to metronidazole [48, 49].

Although mutation is a rare event, the quite growing rapid rate of the bacteria and the absolute number of cells that it reaches favours the quite rapid expression of resistance in a cell population. As a consequence of the mutation spontaneously produced in the bacterial chromosome, once the genes of resistance have been stabilized, they can be directly transferred to all the descendant cells by replicating the DNA, process known as the transfer of genes on the vertical or the vertical evolution [6,10].

During the stress to antibiotics, the saprobiontic bacteria and the pathogenic ones, plainly increase their rate of mutation, meaning they become hypermutant. They express and duplicate the information of survival, also the genes resistant to drugs, situated on plasmids, transposons and integrons [6]. For example, when they are under the stress of antibiotics, some bacteria exchange the genes having a role in the synthesis of some proteinic products, which can increase the rate of mutation inside the bacteria, 10,000 times faster than the rate of mutation that normally appears in the binary cell division. That leads to a kind of hyper–evolution when the mutation acts as a self–defense mechanism for the bacteria, by growing the risk of obtaining an antibiotic resistant mutant [9].

It is worth mentioning the fact that the bacteria have a great capacity to preserve their genetic material conferring them a selective–evolutional advantage and preserves the advantageous mutations even in the presence of the action of the DNA repairing mechanisms, which tend to correct them [6].

Numerous cases of resistance of the pathogen bacterial to macrolides (erythromycin, azithromycin, clarithromycin, dirithromycin, troleandomycin etc. [50]) could be caused by the mutational alteration of the specific nucleotides of the sequence ribosomal RNA (rRNA) 23S of the big ribosomal unity 50S [6]. The adenine 2058 or the adenine adjacent to the linking locus of the peptidil–transferase (A2057 or A2059) is exchanged with another nucleotide by mutation and it confers a high degree of resistance to certain macrolide. A lower level of resistance is produced by the mutations in positions 2057, 2452, 2611, situated outside the centre of interaction of the molecule rRNA with the macrolide. Mutations perturb the structure of the locus of linking the antibiotic to the RNA 23S, hence the decrease of the macrolides' capacity to interact with the ribosomes and to inhibit their activity [6]. The mechanism of this type of mutational resistance has been studied in *Helicobacter pylori*. The infectious agent colonizes the stomach in about 30% of the adult individuals. Most of the infections are asymptomatic, but the *H. pylori* is the main aetiologic agent of most cases of gastric or duodenal ulcers and it is associated with the development of certain types of gastric neoplasia. The treatment of election for the manifest infections is a combination of drugs including a derivative of erythromycin–clarithromycin and an inhibitor of the of the proton pump–omeprazole [50]. The resistance to clarithromycin may appear during therapy [51] and it has been attributed to the mutations in positions A2058 or A2059 of the rRNA 23S. In *H. pylori* there were not identified any genes of methylation of the rRNA or systems of efflux of the macrolide. The mechanisms of resistance seem to be limited by the emergence of mutations rRNA 23S [52]. Considering the very stable sequence of the rRNA in different species of bacteria, it is presumable that the identical mutations will produce the same phenotype in different bacterial species [6].

The rRNA mutations have been rendered evident by the techniques based on PCR [53,54]. The area of the codifying gene for the sequence adjacent to the nucleotide A2058, present in *H. pylori*, was amplified and analyzed by the method of digestion with enzymes of restriction followed by hybridization with oligonucleotide probes with specific sequence and visualization by self–radiography or by nonradioactive techniques. Thus the loci of the ARNr mutation have been identified, which confer resistance to the pathogen bacteria in macrolide [6,53]:

- The emergence of mutations in position A2057 is limited to a group of propionibacteria resistant to erythromycin and to a strain doubly mutant of *H. pylori*, with a mutation at the position 2032, supplementary in comparison with substitution 2057.
- Adenosine 2058 is the essential nucleotide for the interaction of the macrolide with the ribosome. Mutation 2058 to G was the first identified mutation of the ARNr which confers resistance to erythromycin.
- Mutations A2059 to C or G were identified in *Mycobacterium, Propionibacterium, H. pylori* and *Streptococcus pneumoniae*The *in vitro* experiments showed that the mutants A2059 in *H. pylori* have lower levels of resistance to clarithromycin in comparison with the mutants A2058.
